# Carnosic Acid and Carnosol Display Antioxidant and Anti-Prion Properties in In Vitro and Cell-Free Models of Prion Diseases

**DOI:** 10.3390/antiox11040726

**Published:** 2022-04-06

**Authors:** Korina Karagianni, Spyros Pettas, Eirini Kanata, Elisavet Lioulia, Katrin Thune, Matthias Schmitz, Ioannis Tsamesidis, Evgenia Lymperaki, Konstantinos Xanthopoulos, Theodoros Sklaviadis, Dimitra Dafou

**Affiliations:** 1 Department of Genetics, Development and Molecular Biology, School of Biology, Aristotle University of Thessaloniki, 541 24 Thessaloniki, Greece; korinagk@bio.auth.gr (K.K.); spyrospg@bio.auth.gr (S.P.); lioulia@bio.auth.gr (E.L.); 2 Neurodegenerative Diseases Research Group, Department of Pharmacy, School of Health Sciences, Aristotle University of Thessaloniki, 541 24 Thessaloniki, Greece; ekanata@bio.auth.gr (E.K.); xantho@pharm.auth.gr (K.X.); sklaviad@pharm.auth.gr (T.S.); 3 Department of Neurology, German Center for Neurodegenerative Diseases (DZNE), University Medicine Goettingen, 37075 Goettingen, Germany; katrin.thuene@med.uni-goettingen.de (K.T.); matthias.schmitz@med.uni-goettingen.de (M.S.); 4 Department of Prosthodontics, School of Dentistry, Faculty of Health Sciences, Aristotle University of Thessaloniki, 541 24 Thessaloniki, Greece; itsamesidis@auth.gr; 5 Department of Biomedical Sciences, International Hellenic University, 570 01 Thessaloniki, Greece; evlimper@mls.teithe.gr

**Keywords:** Carnosol, Carnosic acid, prion diseases, antioxidant, anti-aggregation, neuroprotection

## Abstract

Prion diseases are transmissible encephalopathies associated with the conversion of the physiological form of the prion protein (PrP^C^) to the disease-associated (PrP^Sc^). Despite intense research, no therapeutic or prophylactic agent is available. The catechol-type diterpene Carnosic acid (CA) and its metabolite Carnosol (CS) from Rosmarinus officinalis have well-documented anti-oxidative and neuroprotective effects. Since oxidative stress plays an important role in the pathogenesis of prion diseases, we investigated the potential beneficial role of CA and CS in a cellular model of prion diseases (N2a22L cells) and in a cell-free prion amplification assay (RT-QuIC). The antioxidant effects of the compounds were confirmed when N2a22L were incubated with CA or CS. Furthermore, CA and CS reduced the accumulation of the disease-associated form of PrP, detected by Western Blotting, in N2a22L cells. This effect was validated in RT-QuIC assays, indicating that it is not associated with the antioxidant effects of CA and CS. Importantly, cell-free assays revealed that these natural products not only prevent the formation of PrP aggregates but can also disrupt already formed aggregates. Our results indicate that CA and CS have pleiotropic effects against prion diseases and could evolve into useful prophylactic and/or therapeutic agents against prion and other neurodegenerative diseases.

## 1. Introduction

Prion diseases or Transmissible Spongiform Encephalopathies (TSEs) are fatal neurodegenerative diseases (NDs) that comprise the various forms of Creutzfeldt–Jakob disease, i.e., sporadic (sCJD), variant (vCJD), and iatrogenic (iCJD), as well as the Gerstmann–Straussler–Scheinker disease (GSS), fatal familial insomnia (FFI), and Kuru, in humans, scrapie in sheep and goats and bovine spongiform encephalopathy (BSE) in cattle. TSEs have long been considered unique among NDs, due to their transmissibility; however, recent research suggests that prion-like features are also present in non-prion NDs [1,2]. Prion diseases typically exhibit a long latency period between infection and clinical manifestation [2,3]. The key event in prion pathogenesis is the structural conversion of the normal, glycolipid-anchored host prion protein, termed PrP^C^, into an abnormally folded conformational isoform, termed PrP^Sc^ [3,4]. PrP^C^ and PrP^Sc^ share the same primary structure, but PrP^Sc^ is β-sheet enriched. This gives rise to further physicochemical and biochemical differences, including reduced PrP^Sc^ solubility in mild detergents, enhanced resistance to partial proteolysis by proteinase K and higher propensity to polymerize into amyloid fibrils [3,4].

The physiological role of PrP^C^ is unknown, but it has been suggested that it is involved in oxidative stress response since it can modulate copper metabolism [4,5] and exerts superoxide dismutase activity [5,6,7]. Furthermore, its conversion to PrP^Sc^ has been associated with oxidative stress in yeast [7,8] and in in vitro models [8,9,10]. In animal models of prion disease, mitochondrial dysfunction, as a result of oxidative stress, has also been reported [10,11,12]. More recently, Nrf2 and the antioxidative response element (ARE) pathway were found to be activated in the cerebellum throughout the presymptomatic and symptomatic stages of a murine model of sCJD and the cerebrospinal fluid of sCJD patients [12,13].

Despite fervent research, key issues, including the mechanisms governing the transmission and spread, as well as the pathogenesis of TSEs, remain elusive, and no effective therapies have been made available as yet [13,14,15]. Identification of novel compounds with prophylactic and/or therapeutic potential against prion diseases thus becomes of crucial importance.

Inhibition of PrP^Sc^ accumulation is considered a primary target for therapeutic intervention. Cell lines infected with TSEs have been routinely used to study prion-related cellular processes and to screen for compounds with potential anti-prion effects [15,16]. Among these, chronically scrapie-infected neuroblastoma cells (N2a22L) have been used extensively as a relevant model for studying TSEs since they are able to promote stable and persistent replication of PrP^Sc^ upon subpassaging [16,17]. More recently, cell-free systems, such as real-time quaking-induced conversion (RT-QuIC) or protein misfolding cyclic amplification (PMCA) have also been employed to evaluate the anti-aggregation properties of compounds [17,18]. Several compounds have been tested for anti-prion activity, including larger molecules such as pentosan polysulfate [18,19], suramin [19,20], amphotericin B [20,21,22], Congo red [22,23], and dendritic polyamines [23,24], and have been shown to possess anti-prion properties. Among small molecules, bis-acridines [24,25], 5,7,8-trimethyl-3,4-dihydro-2H-1,4-benzoxazine derivatives [25,26], polyphenols, phenothiazines, antihistamines, statins, some antimalarial agents [18,19,20,21,22,23,24,25,26,27], indole-3-glyoxamides [28,29], pyridyl hydrazones [29,30], 2-aminothiazoles [30,31], a group of phenethyl piperidines [31,32], and glycodendrimers [32,33] have been shown to inhibit PrP^Sc^ formation in vitro. However, there is still a need for effective compounds when administered before and/or at the onset of neurological symptoms.

Oxidative stress management and metal chelation are also viable but less frequently utilized routes to counter prion diseases (reviewed in [33,34]). In this context, administration of a Mn-SOD2 mimetic with catalase activity led to a statistically significant prolongation of survival time in a murine model of Gerstmann–Sträussler–Scheinker syndrome [34,35]. A case report also indicates that antioxidants may have beneficial effects in prion diseases, as administration of antioxidants was associated with a prolongation of survival of a patient diagnosed with CJD [35,36]. On the other hand, a clinical trial with quinacrine, a molecule that reduces the accumulation of PrP^Sc^ and displays antioxidant effects [24,25], failed to ameliorate the clinical course of the disease [36,37].

**Figure 1:**
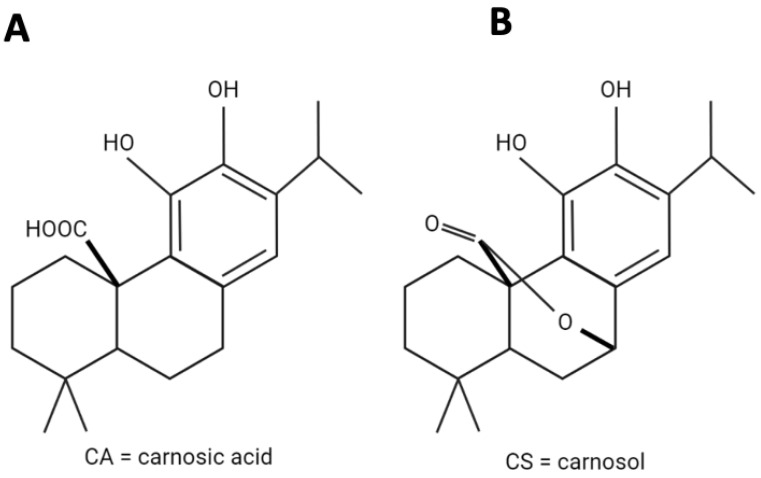
Chemical structures of (**A**) Carnosic acid (MW: 332.43 g/mol) and (**B**) Carnosol (MW: 330.42 g/mol).

Carnosic acid (CA, Figure 1a), a catechol-type electrophilic phenolic diterpene, and its metabolite Carnosol (CS, Figure 1b) are isolated from Rosmarinus officinalis and have antioxidant, anti-inflammatory, neuroprotective, and antineoplastic effects [37,38]. CA binds to specific cysteine residues on Keap1 (Kelch-like ECH-associated protein 1), activating the Keap1-Nrf2 [Keap1-nuclear factor (erythroid-derived 2)- like 2] pathway [38,39]. The Keap1-Nrf2 pathway is one of the main determinants of antioxidant response in cells and through the regulation of several genes, including HMOX1, orchestrates cellular defense mechanisms [39,40], and protects neurons from oxidative stress and excitotoxicity [38,39].

The involvement of oxidative stress in the pathogenesis of prion diseases prompted us to evaluate the antioxidant and anti-prion effect of CA and CS. Our data indicate that both CA and CS have potent antioxidant and anti-prion effects in mouse neuroblastoma cells permanently infected with the mouse-adapted scrapie strain 22L. Furthermore, using the RT-QuiC cell-free system we confirmed that the compounds not only reduce conversion and aggregation of PrP but also disrupt already formed PrP-aggregates. The newly established reduction of PrP^Sc^ accumulation mediated by CA and CS, combined with their well-documented antioxidant effects could be of particular therapeutic relevance for prion diseases, as well as the whole spectrum of NDs, wherein oxidative stress and protein aggregates play a central role in pathogenesis.

## 2. Materials and Methods

### 2.1. Equipment and Reagents

Unless otherwise noted, all materials were obtained from commercial suppliers and used without purification. Minimal essential medium (OptiMEM, 51985042), Dulbecco’s phosphate-buffered saline (DPBS, 14190169), trypsin-EDTA (25200056), fetal bovine serum (FBS, 10270106), and MTT cell viability reagent (M6494) were purchased from Invitrogen (Eugene, OR, USA). Proteinase K was purchased from Merck (1.24569.0100, Darmstadt, Germany). Monoclonal anti-PrP antibody 6H4 was a generous gift from Thermo Fisher Scientific/Prionics (7500997, Waltham, MA, USA), and monoclonal anti-actin antibody β-actin (C4) was obtained from Santa Cruz Biotechnology (47778). CDP star Western blotting substrate was from NEB (870115), and Bradford solution (A6932,0250) was from AppliChem. RNA purification kit was from Macherey Nagel (Nucleospin RNA II, 740955.250), PrimeScript cDNA synthesis kit was purchased from TAKARA (RR037A), and KAPA SYBR fast qPCR kit from KAPA Biosystems (KK4601). recPrP^C^ for the RT-QuIC assay was from Thermo Fisher Scientific/Prionics, the black optical bottom plates were from Fisher-Scientific, and the FLUO Star OPTIMA plate reader used to incubate and read RT-QuIC reactions was from BMG Labtech. A Tecan microplate reader (model 5200220) was used to measure the fluorescence of oxidized H_2_DCFDA (Invitrogen, D399) and a BIOBASE-EL 10A microplate reader to estimate optical density. Carnosic acid (C0609), Carnosol (C9617), Thioflavin-T (T3516), and all other chemical reagents were purchased from Sigma-Aldrich.

### 2.2. Cell Culture

The murine neuroblastoma N2a58 cell line, overexpressing murine PrP, and the corresponding scrapie-infected cell line, propagating the 22L mouse-adapted scrapie strain (N2a22L) [16], were used throughout the study. Both cell lines were a generous gift from Prof. Sylvain Lehmann, Centre Hospitalier Universitaire de Montpellier, France.

Handling of infectious material was performed in a biosafety level 2 facility. Cells were cultured in Opti-MEM, supplemented with 10% (*v*/*v*) FBS in a 5% CO_2_ incubator maintained at 37 °C. Media was changed every 3 days, and subpassaging of the cells was performed every 4 (N2a58) or 5 (N2a22L) days. All experiments were performed in Mycoplasma-free cultures.

### 2.3. LD_50_ Estimation

The LD_50_ of CA and CS was estimated in N2a22L and N2a58 cells using 3-(4,5-dimethylthiazol-2-yl)-2,5-diphenyl-2H-tetrazolium bromide (MTT) Assay, following incubation of the cells in increasing amounts of the compounds [40,41]. CA and CS were added to the culture medium in a range of concentrations (10, 20, 30, 40, 50, 60, 70, 80, 90, 100, and 150 μM) and incubated for 48 h. Since CA and CS were initially diluted in dimethyl sulfoxide (DMSO), control cell cultures received DMSO only in concentrations matching the final concentration of DMSO delivered with the compound in each well. In all cases, DMSO concentration did not exceed 0.1% *v*/*v*. Each condition was tested in triplicates.

Following incubation, a 10% *w*/*v* solution of MTT was added, and the cultures were further incubated for 4 h (37 °C, 5% CO_2_). OD_570_ and correction OD_630_ were read in a microplate reader immediately after incubation. Absorbance readings were transformed to survival rates by measuring the absorbance reading of the control cell cultures and assigning the 100% survival rate to the control cell cultures. The survival rates were plotted against the concentration of the compounds, and the curves were prepared. LD_50_ was the concentration of the compound, leading to a 50% survival of the cells.

### 2.4. Fluorescence Assay

For the detection of reactive oxygen species (ROS), the cell-permeable ROS-sensitive probe 2′,7′-dichlorodihydrofluorescein diacetate (H_2_DCFDA), which fluoresces at 520 nm (λex 480 nm) upon oxidation, was used [42]. N2a58 and N2a22L cells were plated in 96-well plates (2500 cells/well) for 24 h and incubated with CA and CS for 48 h. Control cells were incubated in matching concentrations of DMSO. Following incubation with CA and CS, H_2_DCFDA (prepared as a 0.5 mM stock solution in DMSO) was added to the cultures at a final concentration of 20 μΜ, and the cells were further incubated for 30 min at room temperature. To eliminate the effect of autofluorescence, CA- and CS-treated N2a58 and N2a22L cells were also incubated with DMSO alone. Oxidation of H_2_DCFDA was monitored by measurement of the fluorescence in the cell supernatant in black microplates using a Tecan fluorometer. In a different set of experiments, prior to incubation with CA and CS, cells were treated with 600 μM H_2_O_2_ for 30 min to induce oxidative stress. The amount of ROS generated was estimated with H_2_DCFDA, as described. In all cases, the relative fluorescence was expressed as “% maximal emission”, as determined with the Tecan Magellan software (https://lifesciences.tecan.com/software-magellan (access on 1 March 2022)). Maximal emission was defined as the fluorescence emission obtained from a reaction in which only H_2_DCFDA and H_2_O_2_ (at a final concentration of 3 mM) were added. The H_2_O_2_ concentration in this reaction was selected after preparing a standard curve with different H_2_O_2_ concentrations ranging between 0.1 and 5 mM and recording the fluorescence. H_2_O_2_ at a final concentration of 3 mM produced maximal fluorescence readings without overflow.

### 2.5. RNA Isolation

N2a58 and N2a22L were incubated in the presence of CA and CS for 48 h and then total RNA was extracted using Macherey Nagel Nucleospin RNA II columns, following the manufacturer’s protocol. The purified RNA was quantified spectrophotometrically using Nanodrop 2000 (Thermo Scientific, Waltham, MA, USA). cDNA synthesis was performed using TaKaRa PrimeScriptTM RT reagent kit (Protocol: Reverse transcription) starting with 500 ng of total RNA.

### 2.6. qPCR

Expression levels of the genes encoding for PrP (*PRNP,* Accession number: NC_000068.8, Gene ID: 19122), Heme oxygenase 1 (*HMOX1,* Accession number: NC_000074, Gene ID: 15368), Nuclear factor (erythroid-derived 2)-like 2 (*NFE2L2,* Accession number: NC_000068.8, Gene ID: 18024), Glutamate-cysteine ligase regulatory subunit (*GCLM*, Accession number: NC_000069, Gene ID: 14630) and β-Actin (*ACTB*, used as a reference gene, Accession number: NC_000071.7, Gene ID: 11461), were estimated by real-time quantitative PCR (qPCR), using 20 ng cDNA as a template. Each sample was analyzed in triplicates. Primer sequences for the amplification of gene fragments of interest are given in Table 1. qPCR was performed in the 7500 Fast Real-time PCR system (Applied Biosystems) using the KAPA SYBR fast qPCR kit. After initial denaturation for 20 s at 95 °C, the following cycle was repeated 40 times: 3 s at 95 °C, 30 s at 60 °C, 25 s at 72 °C, and fluorescence estimation at 80 °C. Accumulation of PCR product was monitored by measurement of SYBR Green I emission at the end of the extension step after each cycle.

### 2.7. Inhibition of PrP^Sc^ Accumulation in Cell Lines

Cells were treated with CA and CS for 48 h. Since CA and CS were added in the cell culture diluted in DMSO, control cells received DMSO only at the same concentrations as cells receiving the compounds. All experiments were carried out in 3 independent experiments with biological replicates (separate passaged cells, within 5 passage numbers in total), and each biological replicate was tested in 3 technical replicates. Immediately before cell lysis, cells were checked for toxic effects, bacterial contamination, and cell density by light microscopy.

Prior to trypsinization, cells were rinsed once in PBS and then incubated with 0.25% *w*/*v* trypsin for 5 min (37 °C, 5% CO_2_). Cells were then rinsed with PBS and incubated with ice-cold lysis buffer (10 mM Tris (pH 7.5), 100 mM NaCl, 10 mM EDTA, 0.5% Triton-X-100 *v*/*v*, 0.5% sodium deoxycholate *w*/*v*), for 10 min on ice. The lysed cells were centrifuged for 1 min at 14,000× *g* to remove cell debris, and the supernatant, which contained whole proteome lysate, was used for further analysis.

A small fraction of the preparation was used for protein content estimation and SDS-PAGE/Western blotting following methanol precipitation. To this end, phenylmethylsulfonyl fluoride (PMSF, 5 mM final concentration) and 9 x volumes of ice-cold methanol were added, and the preparation was incubated for 24 h at −80 °C. Proteins were recovered by centrifugation at 15,000× *g* for 30 min at 4 °C and either suspended in PBS for protein content estimation using the Bradford reagent, following the manufacturer’s protocol or diluted in O’Farrell buffer (50 mM Tris pH 6.8, 2% SDS (*w*/*v*), 3% β-mercaptoethanol (*v*/*v*), 10% Glycerol (*v*/*v*)), heated to 95 °C for 5 min, briefly centrifuged and analyzed by SDS-PAGE and Western Blot. A second lysate fraction was treated with proteinase K (PK) to digest PrP^C^ and allow detection of the PK-resistant PrP^Sc^ present in the sample. For PK treatment, cell lysates were incubated with PK (1.25 μg PK/mg total protein) for 1 h at 37 °C after the addition of lauryl-sarcosyl 1% *w*/*v*. The digestion was stopped by the addition of PMSF, and the proteins were methanol-precipitated and prepared for SDS-PAGE and Western blotting as previously described.

For SDS-PAGE [43,44], 100 μg (PK-treated samples) and 50 μg (non-PK treated samples) of total protein were resolved on 12% *w*/*v* polyacrylamide gels. Following electrophoresis, proteins were electrotransferred onto Polyvinylidene Fluoride (PVDF) membranes using a mini-transblot cell for 2 h at 100 V at 4 °C. Membranes were blocked in blocking buffer (5% *w*/*v* non-fat dry milk in PBS containing 0.1% *v*/*v* Tween 20 (PBST)) for 1 h at room temperature (RT) and then incubated with 6H4 (0.2 µg/mL in blocking buffer, overnight at 4 °C) and an AP-conjugated rabbit anti-mouse IgG (0.1 µg/mL in blocking buffer, RT, 1 h). PrP was visualized on X-ray films using CDP-star. The same membrane was used for estimation of actin levels, using a β-actin (C4) antibody (0.1 µg/mL in blocking buffer, RT, 1 h) and an AP-conjugated rabbit anti-mouse IgG (1:5000, 1 h RT) following a brief (10 min) wash with PBST. CDP-star was used for the visualization of the protein bands.

Normalization of protein levels was based on actin levels. Protein content was estimated densitometrically using ImageJ software (freely available at http://rsb.info.nih.gov/ij/, Access Date: 3 May 2020), following the digitization of the X-ray films with a flatbed scanner, a widely accepted protocol. Multiple exposures of each image were assessed to ensure the readouts were always within the linear dynamic range of the film.

### 2.8. RT-QuIC

Real-time quaking-induced conversion (RT-QuIC) reactions were performed in triplicates, as previously described [43]. In brief, 15 μL of cerebrospinal fluid (CSF, diluted 1000 fold), originated from the National Reference Center for TSE, Goettingen, Germany, from 4 different RT-QuIC positive patients with a confirmed sCJD diagnosis (*PRNP* codon 129 genotype MM) were mixed with 85 μL of reaction buffer consisting of 5× PBS (pH 6.9), 170 mM sodium chloride, 1 mM EDTA, 10 μM Thioflavin-T and 0.1 mg/mL recPrP^C^. CA and CS were diluted in DMSO and added to a final concentration of 1 mM either at the beginning of the reaction or after aggregates were formed (approximately 40 h later), to evaluate inhibition or disruption of aggregation, respectively. The reactions were setup in 96-well black optical bottom plates and carried out in a BMG Labtech FluoO Star OPTIMA plate reader at 42 °C for 80 h with intermittent quaking cycles (double-orbital quaking at 600 rpm: 60 s, incubation break: 60 s). Kinetics of the beta-sheet formation were monitored by estimating Thioflavin-T fluorescence (excitation 450 nm, emission 480 nm) every 30 min.

### 2.9. Statistical Analyses

The GraphPad Prism version 8.0.2 for Windows (GraphPad Software, San Diego, CA, USA, www.graphpad.com, Access Date: 29 April 2019) was used for statistical analyses. Non-linear regression analysis using dose-response equations was used to achieve curve fitting and determine LD_50_ values for the tested compounds in each cell line. Unpaired, one-tailed *T*-test analysis was performed to evaluate the statistical significance of phenotypic differences (PrP^Sc^ accumulation, gene expression alterations) between untreated and treated cells. *p* values lower than 0.05, were considered statistically significant.

## 3. Results

### 3.1. Assessment of LD_50_ of Carnosic Acid (CA) and Carnosol (CS) in N2a58 and N2a22L Cells

In this study, we used 22L-scrapie-infected mouse neuroblastoma cells (N2a22L), a well-established in the field [25,26,44] in vitro prion disease model, to evaluate the anti-prion effects of Carnosic acid and Carnosol. We first assessed the potential toxic effects of the compounds on the cell lines used in the study by incubating the cells for 48 h in the presence of different concentrations of the compounds and estimating the survival rates using the ΜΤΤ assay. Εxperiments were carried out in three independent experiments, including appropriate technical replicates and biological controls. Survival curves were plotted, and the LD_50_ (50% lethal concentration) was calculated for each compound in each cell line (Supplementary Figure S1). Toxicity levels were comparable for CA in both N2a58 and N2a22L cells (LD_50_: 29.68 and 27.15 μΜ, respectively), whereas CS was more toxic in N2a22L (LD_50_: 25.70 μΜ) compared to N2a58 (LD_50_: 33.06 μΜ) cells (Supplementary Figure S1). Based on the toxicity assessment, the effects of CA on N2a58 cells were estimated at a concentration of 14.84 μM and on N2a22L at a concentration of 13.58 μM, whereas effects of CS were estimated at final concentrations of 16.53 and 12.85 μM for N2a58 and N2a22L cells, respectively. These concentrations correspond to half the LD_50_ (0.5 × LD_50_) for each compound and cell line. In all cases, cells were incubated with the compounds for 48 h.

### 3.2. Evaluation of Antioxidant Effect of Carnosic Acid (CA) and Carnosol (CS) in N2a58 and N2a22L Cells

**Figure 2:**
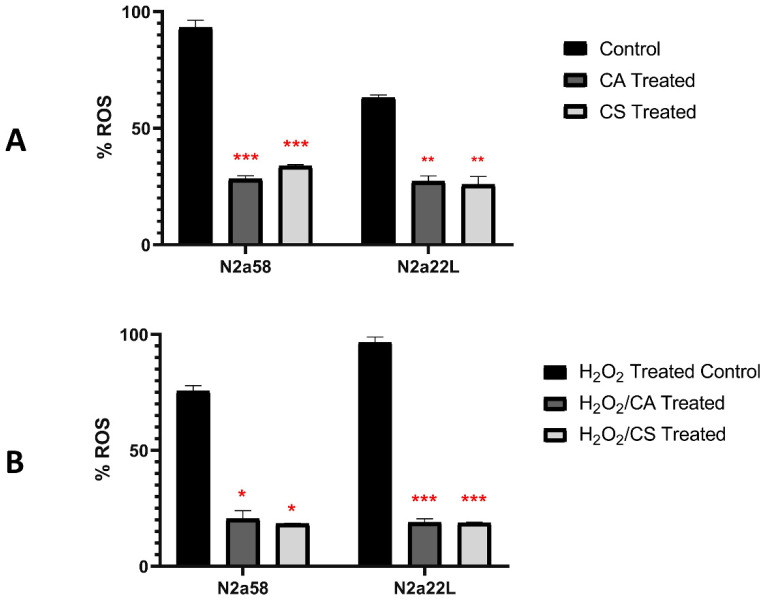
Reactive oxygen species (ROS) measured using H_2_DCFDA in N2a58 and N2a22L cell lines after treatment with the 0.5 × LD50 of Carnosic acid (CA) and Carnosol (CS) without (**A**) or following (**B**) pre-treatment with 600 μΜ H_2_O_2_ (2 replicates for each condition). 0.5 × LD50 was estimated at 14.84 μΜ for CA and 16.53 μΜ for CS for N2a58 and at 13.58 μΜ CA and 12.85 μΜ CS for N2a22L. The % ROS was calculated based on the maximum ROS production value (3 mM). Data represent means ± SD; stars denote statistical significance (unpaired, one-tailed, *T*-test); *: *p*-value < 0.05, **: *p*-value < 0.01, ***: *p*-value < 0.001. CA: dark grey, CS: light grey, Control: black.

To assess the antioxidant effect of CA and CS in N2a58 and N2a22L we measured the amount of reactive oxygen species (ROS) produced in the cells with or without pre-treatment with the compounds using the ROS-sensitive fluorescent probe H_2_DCFDA [42]. The amount of ROS was first evaluated in the presence of 0.5 × LD_50_ of each compound and cell line (14.84 μΜ CA and 16.53 μΜ CS for N2a58 and 13.58 μΜ and 12.85 μΜ, respectively, for N2a22L cells (Figure 2). The production of ROS was significantly decreased in the presence of CA and CS for both cell lines by more than 60% in comparison to the untreated cells (*p*-value < 0.0021).

To further confirm the antioxidant properties of CA and CS, both cell lines were pretreated with H_2_O_2_ (600 μΜ) to induce oxidative stress-mediated damage. As expected, H_2_O_2_ induced both cell death and ROS production (data not shown), while CA and CS significantly decreased the ROS levels in comparison to H_2_O_2_-treated cells (*p*-value < 0.0002). 

### 3.3. Effects of Carnosic Acid and Carnosol on Gene Expression

**Figure 3:**
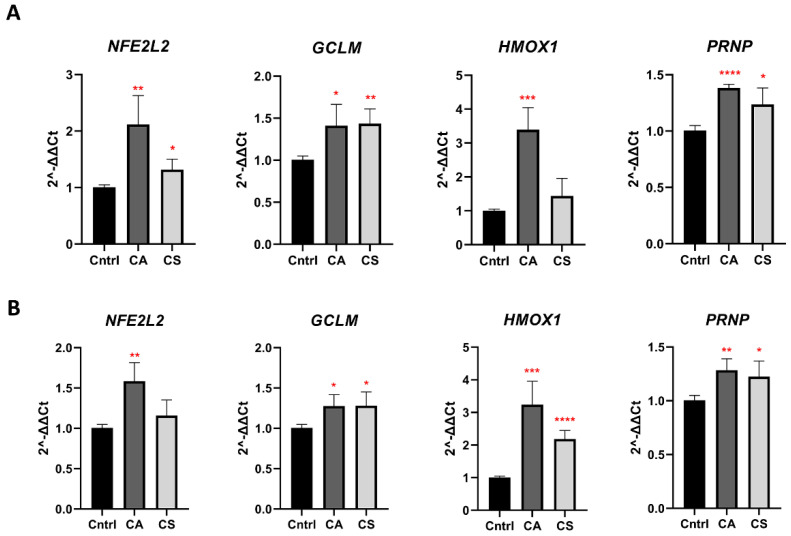
Treatment with CA and CS upregulates Keap-Nrf2 regulated genes and *PRNP*. qPCR for the expression of genes regulated by the antioxidant response Keap-Nrf2 pathway and *PRNP* was performed using mRNA extracted from N2a58 (**A**) and N2a22L (**B**) cells treated with 0.5 × LD_50_ of CA or CS for 48 h and compared to non-treated control cells (Cntrl). 0.5 × LD50 was estimated at 14.84 μΜ for CA and 16.53 μΜ for CS for N2a58 and at 13.58 μΜ CA and 12.85 μΜ CS for N2a22L. Data represent means ± SD of three independent experiments. Stars denote statistical significance (unpaired, one-tailed, *T*-test); *: *p*-value < 0.05, **: *p*-value <0.01, ***: *p*-value < 0.001, ****: *p*-value < 0.0001. CA: dark grey, CS: light grey, Control: black.

Electrophilic compounds often mediate their neuroprotective and antioxidant effects through activation of the Keap1-Nrf2 pathway. The binding of an electrophilic compound to C151, C273, and C288 in the human Keap1 protein is the initial reaction of the activation cascade [45], leading to upregulation of the transcription of phase 2 enzymes, including Heme oxygenase 1 (*HMOX1*), and the modifier subunit of Glutamate-cysteine ligase (*GCLM*). Many of these enzymes are involved in the cellular redox response and their upregulation provides protection against oxidative stress. We thus examined whether incubation of N2a58 and N2a22L cells with CA and CS would upregulate the expression of *NFE2L2* (encoding for Nrf2), HMOX1, and GCLM, further contributing to the antioxidant response. Furthermore, we evaluated the expression level of *PRNP*, encoding for PrP^C^ (Figure 3).

Treatment of N2a58 and N2a22L cells with 0.5 × LD_50_ CA led to a statistically significant upregulation of *NFE2L2, HMOX1,* and *GCLM*, indicating that CA promotes the expression of enzymes involved in the antioxidant response, thus further protecting the cell from oxidative insults. In this case, upregulation of *HMOX1* was more marked, while upregulation of *NFE2L2* and *GCLM* were more modest compared to control cells. 

When N2a58 and N2a22L cells were treated with CS at 0.5 × LD_50_ a similar trend for upregulation of *NFE2L2, HMOX1,* and *GCLM* was observed. The upregulation induced by CS was, in most cases, less marked than CA, especially for *NFE2L2* and *HMOX1*, which did not reach statistical significance in N2a58 and N2a22L cells, respectively. Interestingly, *PRNP* expression increased upon treatment of both cell lines with both compounds.

### 3.4. Carnosic Acid and Carnosol Reduce PrP^Sc^ Levels in In Vitro Models of Prion Diseases

We next evaluated the potential effect of CA and CS on the conversion of PrP^C^ to PrP^Sc^, using N2a22L cells, a well-characterized cellular model in which PrP^C^ is constitutively converted to PrP^Sc^. Upon conversion to PrP^Sc^, the protein acquires a different structure, providing partial resistance against proteolysis by proteinase K. Since PrP^Sc^-specific antibodies are not available, we used this trait to decorate PrP^Sc^ in cell extracts, following incubation with the compounds. To this end, N2a22L cells were treated with 0.5 × LD_50_ of the compounds or DMSO and the cell lysates were divided in two fractions: the first fraction was treated with PK, while the second fraction was not. The fractions were then prepared for Western blotting and the PK-treated fraction was immunolabelled for PrP, while the PK was untreated for PrP and actin.

To confirm that the conditions used for the proteinase K treatment led to full digestion of PrP^C^, cell extracts from the non-prion infected N2a58 cells were similarly treated with PK, and the extracts immunolabelled for PrP. Since no PrP immunoreactivity was detected in these experiments (Supplementary Figure S2), we deduced that the PK treatment protocol used for the analysis completely digested PrP^C^ and that the PK-treated immunoreactive bands detected in N2a22L cells correspond to PrP^Sc^ and not to partially digested PrP^C^.

For each sample, we estimated the ratio of PK resistant fraction versus total *PrP* using the following formula:

PrP RESPrP TOTActin TOT

where *PrP_RES_* is the intensity of the *PrP* bands in the PK-treated fraction; *PrP_TOT_* is the combined intensity of the *PrP* bands in the non-PK-treated fraction; and *Actin_TOT_* is the intensity of the actin band in the non-PK treated fraction.

We then computed the conversion efficiency for each CA or CS-treated sample, by dividing the values obtained from the above calculation with the calculated value of the DMSO-treated sample. To rule out the effect of antibodies titer, substrate, and film development reagents, as well as different exposure times, pK-treated and untreated samples from cells cultured in the presence of CA, CS or DMSO were resolved on the same blot and each data point was calculated using densitometry data originating from the same exposure of the blot.

**Figure 4:**
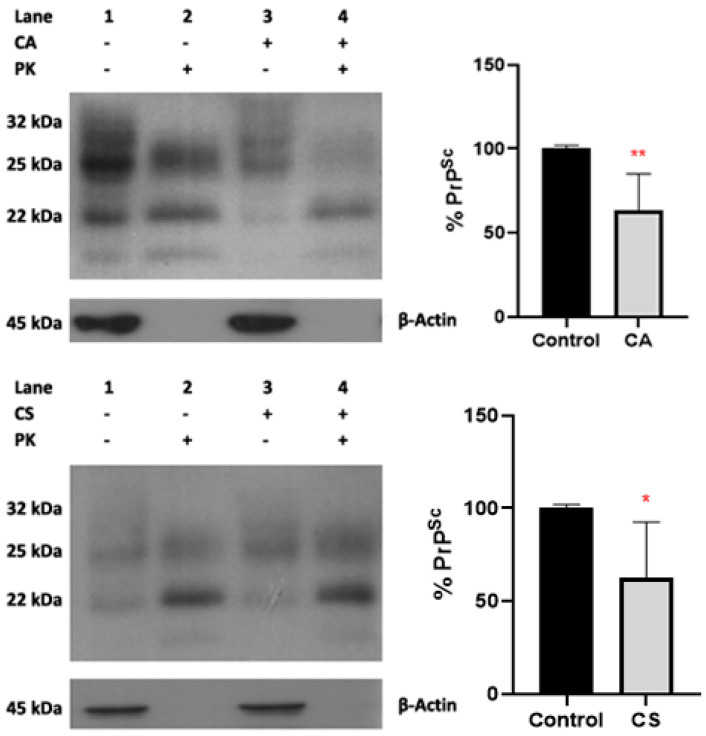
Effect of Carnosic acid (CA) and Carnosol (CS) treatment on PrP^Sc^ levels (in vitro assays). N2a22L cells were incubated with 0.5 × LD_50_ of CA and CS for 48 h or left untreated (Control), lysed, and then divided into two different fractions. 0.5 × LD_50_ was estimated at 14.84 μΜ for CA and 16.53 μΜ for CS for N2a58 and at 13.58 μΜ CA and 12.85 μΜ CS for N2a22L. Fraction lysates were either treated (+) or not (-) with PK, and 50 μg of PK-untreated or 100 μg of PK-treated fraction was resolved by SDS-PAGE and immunoblotted with the anti-PrP antibody 6H4 (1:5000) and an anti-β-actin (1:2000) antibody. One representative blot for each compound, along with the densitometric analysis from three independent experiments, are depicted. Data represent means ± SD; stars denote statistical significance (unpaired, one-tailed, *T*-test); *: *p* value < 0.05, **: *p* value < 0.01. CA: dark grey, CS: light grey, Control: black.

Treatment with CA and CS led to a statistically significant reduction of the PrP^Sc^ signal by approximately 40% in N2a22L cells (Figure 4).

### 3.5. Carnosic Acid and Carnosol Inhibit de Novo Formation of PrP^Sc^ Aggregates and Disrupt Already Formed Aggregates

**Figure 5:**
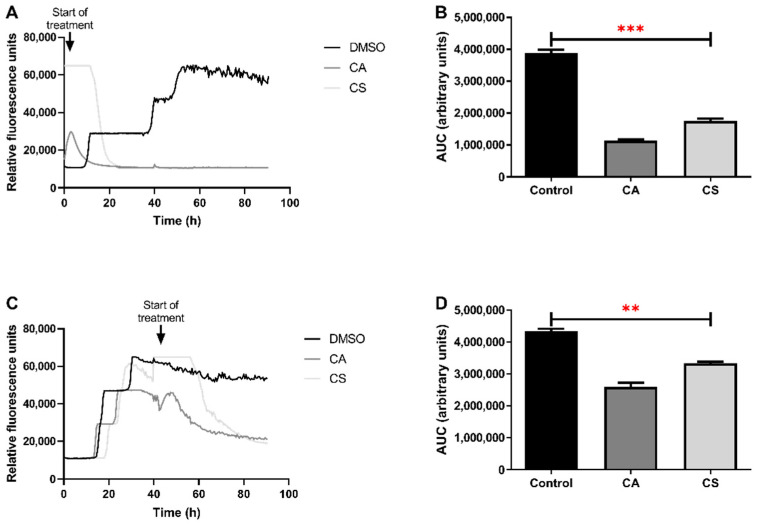
RT-QuIC assays. Aggregation of recPrPC in RT-QuIC assays using CSF from four different sCJD patients as a seed was evaluated in the presence or absence of CA and CS (1 mM final concentration). (**A**) CA and CS were added to the reactions from the beginning, and the Th-T fluorescence was recorded every 30 min. In control reactions containing only DMSO, the solvent vehicle for the compounds, PrP^Sc^ aggregates are formed, whereas CA and CS block their formation. (**B**) AUC values, estimating Th-T fluorescence (and thus aggregation) of each reaction were computed, and the statistical significance was estimated by *T*-test. The addition of CA led to a reduction of protein aggregation by ~71%, whereas CS-induced reduction was ~55%. (**C**) RT-QuIC assays were started without the addition of the compounds, and Th-T fluorescence reached a plateau, indicating conversion and aggregation of PrP^Sc^. The addition of CA and CS to the reaction mixture led to a pronounced decline in Th-T fluorescence. (**D**) AUC values were estimated for the reactions and a statistically significant reaction of the protein aggregation by ~40% for CA and ~23% for CS was observed. Graphs (**A**,**B**) depict data from reactions using CSF from four different sCJD patients as a seed (each reaction performed in triplicates). Columns in (**B**,**D**) represent means ± SD of the AUC calculated for the individual fluorescence curves of each replicate reaction; stars denote statistical significance (unpaired, one-tailed, *T*-test); **: *p* value < 0.01, ***: *p* value < 0.001. CA: dark grey, CS: light grey, Control: black.

To better understand the effects of CA and CS in prion aggregation we performed RT-QuIC assays, using pooled CSF from patients with sCJD as the seed and monitored conversion and aggregation of recombinant PrP^C^ (recPrP^C^), via Thioflavin-T (Th-T) fluorescence. Two types of assays were performed: to study the effect of CA and CS in the de novo conversion of PrP^C^ to PrP^Sc^ and the formation of aggregates; we added the compounds diluted in DMSO from the beginning of the assay at a final concentration of 1 mM. In this setting, both CA and CS prevented conversion of recPrP^C^ to PrP^Sc^, as evidenced by the lack of Th-T fluorescence 40 h after the assay had started. Conversely, in control reactions, in which only DMSO was added at a final concentration equal to its concentration in the reactions with the compounds (2% *v*/*v*), aggregates were generated, leading to Th-T fluorescence (Figure 5A). To better estimate the effect of the compounds, we calculated the area under the curve (AUC) for each reaction as a measure of protein conversion and aggregation. AUCs for both reactions containing the compounds were significantly lower, by ~71% for CA and ~55% for CS compared to the control reaction (Figure 5B). To exclude possible non-specific effects associated with the experimental setup, further control reactions were prepared in the absence or presence of DMSO (2% *v*/*v*) or using CSF samples from non-CJD patients or without the addition of recPrP^C^ in the reaction. These controls reactions confirmed that the formation of aggregates is only possible in the presence of recPrP^C^ and CSF from CJD patients as a seed and that DMSO does not have a significant effect in the conversion, further validating our findings (Supplementary Figure S3).

To study the effects of the compounds on already formed aggregates, similar reactions were setup, but CA and CS, at a final concentration of 1 mM, were added 40 h after the reactions had started. In this setting, PrP^C^ had already been converted to PrP^Sc^ and aggregates were present in the reaction mix, as evidenced by the plateau in Th-T fluorescence (Figure 5C). The addition of CA and CS to the reactions led to a short-term reduction in Th-T fluorescence, associated with the dilution of the aggregates, followed by fast recovery due to the formation of new aggregates. However, further incubation for approximately 10 (in the case of CA) or 20 (in the case of CS) hours with the compounds led to a steady decline in Th-T fluorescence, arising from the disruption of the already formed aggregates brought about by the compounds. As expected, in control reactions wherein only DMSO had been added, a similar reduction in Th-T fluorescence reading was not observed (Figure 5C). These findings were further confirmed by the AUC values estimated, which showed a significant reduction of ~40% and ~23% when CA and CS were added (Figure 5D).

## 4. Discussion

Despite the weak evidence for the direct neurotoxicity of PrP^Sc^, there are numerous reported detrimental effects of PrP^Sc^ formation/aggregation that explain at least some of the toxicity of these complexes. This includes the induction or inhibition of a range of cellular processes, such as autophagy, induction of apoptosis, proteasome/ubiquitin inhibition, synaptic dysfunction, and oxidative stress (reviewed in [45,46]). It is unclear whether oxidative stress results from loss of PrP^C^ function and/or is a driver of pathogenesis. PrP^C^ is involved in antioxidant responses via modulation of copper metabolism [4,5] and intrinsic superoxide dismutase activity [5,6,7]. Accordingly, oxidative stress has been associated with the conversion of PrP^C^ to PrP^Sc^ in yeast and in in vitro studies [7,8,9]. Alterations in free radical metabolism and increased oxidative stress can cause mitochondrial dysfunction in the CNS of scrapie-infected animals, suggesting that mitochondrial dysfunction is a contributing factor to prion disease progression [46,47]. It is well-documented that overproduction of reactive oxygen species (ROS) generates oxidative stress in cells and that oxidative stress results in various pathophysiological conditions, including prion diseases [47,48]. The Keap1-Nrf2 regulatory pathway plays a central role in protecting cells against oxidative and xenobiotic stresses. The Nrf2 transcription factor upregulates the expression transcription of several cytoprotective genes that have been implicated in protection from NDs, suggesting that Keap1-Nrf2 system is a potential target of therapeutic intervention against NDs [48,49].

Electrophilic compounds are a newly recognized class of redox-active neuroprotective compounds with electron-deficient, electrophilic carbon centers that react with specific cysteine residues on targeted proteins via thiol (S-)alkylation. Although plants produce a variety of physiologically active electrophilic compounds, their detailed mechanism of action remains unknown. Catechol ring-containing compounds have attracted attention because they become electrophilic quinones upon oxidation, although they are not themselves electrophilic. Carnosic acid and its metabolite carnosol have been isolated from Rosmarinus officinalis and activate the Keap1-Nrf2 transcriptional pathway by binding to specific Keap1 cysteine residues, protecting in this way neurons from oxidative stress and excitotoxicity [38,39,40,44,45,46,47,48,49,50,51,52].

Given the association between prion diseases and other NDs with oxidative stress, we evaluated the antioxidant and anti-prion effects of CA and CS in N2a22L cells, a widely used in vitro model. As anticipated, CA and CS significantly reduced the ROS burden in both N2a22L cells and their non-prion infected counterparts, N2a58, even when ROS production was amplified following incubation with H_2_O_2_. In the case of CA, these effects were at least in part associated with the upregulated expression of the genes encoding for the transcription factor Nrf2 and some phase 2 enzymes, whose expression is controlled by Nrf2. When cells were treated with CS, upregulation of the genes encoding for Nrf2 and the phase 2 enzymes was also achieved; however, for some genes (*NFE2L2* in N2a22L and *HMOX-1* in N2a58 cells), the upregulation did not reach statistical significance. It should be noted that subcellular localization of Nrf2 plays a major role in its function and shifting from the cytoplasm to the nucleus drives the expression of many genes. On top of expression upregulation, treatment with CA and CS could facilitate the migration of Nrf2 to the nucleus, thus increasing the expression of the phase 2 enzymes.

In addition to the antioxidant effects, incubation of N2a22L cells with CA and CS led to a significant reduction of the PrP^Sc^ burden, as evidenced by the statistically significant reduction of the normalized PrP^Sc^ immunoreactivity in PK treated fractions of N2a22L cell lysates. Treatment of the cells with CS also resulted in a reduction of PrP^Sc^ accumulation; however, the reduction was smaller and did not reach statistical significance.

To better understand whether the reduction in PrP^Sc^ accumulation is associated with the antioxidant effects, a reduction of PrP^Sc^ formation or an enhancement of PrP^Sc^ clearance, we studied the effects of CA and CS in the conversion of recombinant PrP^C^ to PrP^Sc^ in a cell-free system, using the RT-QuIC assay. In these experiments, CA and CS inhibited *de novo* generation and aggregation of PrP^Sc^, indicating that the observed reduction of PrP^Sc^ accumulation in the prion-infected cells is the result of direct inhibition of PrP^Sc^ generation and that the antioxidant effects or the enhanced clearance are secondary mechanisms, as far as PrP^Sc^ accumulation is concerned. RT-QuIC assays, revealed that CA and CS not only inhibit *de novo* formation of PrP^Sc^, but also disrupt already formed PrP^Sc^ aggregates. This effect is of particular importance in prion diseases, which are characterized by a long asymptomatic period following infection, as it implies beneficial effects even if their administration started after the appearance of clinical symptomatology, which is typically associated with elevated PrP^Sc^ aggregates burden.

Although formal dose-response assays were not performed for the anti-prion effects of CA and CS, results from the RT-QuiC assays appear to agree with the in vitro assays, since the effects of CA are more prominent than CS. Preliminary results in which N2a22L cells were treated with higher concentrations of CS show further reduction of PrP^Sc^ accumulation at the expense of marginally reduced cell viability (data not shown). Identification of the exact molecular mechanisms underlying the inhibition of *de novo* PrP^Sc^ formation by CA and CS are underway and could enable further improvement of the anti-prion effects through consideration of the structure–activity relationships. CA has been previously associated with the reduction of the production of amyloid-β 1–42 in SH-SY5Y cells. However, in this case, the reduction was associated with overexpression of the gene encoding for the metalloprotease ADAM17 [49]. Our results from the cell-free systems indicate a direct effect of CA and CS in the formation of PrP^Sc^.

Administration of CA and CS led to an upregulation of *PRNP*, the gene encoding for the prion protein, probably mediated through activation of the Keap1-Nrf2 pathway. An Nrf2 binding site has already been identified at the *PRNP* promoter; however, as opposed to this study, overexpression of Nrf2 lead to a reduced activity of the *PRNP* promoter [50,52]. The apparent discrepancy between our results and previous results could be attributed to the different experimental setup used in the study by Cichon et al., where luciferase assays were performed to study the activity of the *PRNP* promoter upon overexpression of Nrf2. On the other hand, we used an activator of the Keap1-Nrf2 axis and estimated *PRNP* levels via quantitative PCR. Interestingly, Cichon et al. reported elevated PrP^C^ protein levels when Nrf2 was overexpressed.

Administration of CS also led to an increase of PrP^C^, in agreement with qPCR data and the findings by Cichon et al. Following administration of CA, PrP^C^ protein level remained stable, despite upregulation at the mRNA level. It should be noted that transcription and translation do not always follow a linear regression, and different parameters may influence the dynamic properties of either transcription or translation into protein. Several mechanisms can repress the synthesis of proteins from a certain copy number of mRNA molecules, including factors affecting mRNA and protein ratio, such as regulatory proteins and RNA editing [52,53]. Considering our findings and the already published data on Nrf2-mediated control of PrP^C^ expression, it would be safe to assume that regulation of PrP^C^ expression via the Keap1-Nrf2 axis is more complex than anticipated and possibly different regulatory mechanisms fine-tune the process.

## 5. Conclusions and Future Perspectives

Our data indicate that Carnosol and Carnosic acid display potent antioxidant effects, block the conversion of PrP^C^ to PrP^Sc^ and its concomitant aggregation, and can disrupt the already formed PrP^Sc^ aggregates. Remarkably, it has been experimentally demonstrated that Carnosic acid can penetrate the blood–brain barrier and reach the brain parenchyma after oral administration [39]. Likewise, using the LightBBB computational prediction model [54] it seems that Carnosol can also penetrate the blood-brain barrier, yet this has not yet been experimentally confirmed. This rare combination of effects could render the compounds particularly useful for prophylaxis and for the treatment of prion diseases. Further in vivo studies are underway to better characterize their efficiency in TSEs and other NDs. We aim to utilize RML mice, a stable mouse-adapted scrapie model, as well as a humanized CJD mouse model (Tg340) that faithfully recapitulates the CJD molecular and neuropathological phenotype, to further validate their prospective protective and therapeutic value.

## Figures and Tables

**Table 1:** Primer sequences used for the amplification of fragments from genes encoding murine PrP (PRNP), murine Nrf2 (NFE2L2), murine heme oxygenase 1 (Hmox), murine c-glutamyl-cysteine-ligase (Gclm), and ACTB (β-Actin, reference gene).

Primer	Gene	Sequence (5′-3′)	Amplified Fragment (bp)
qRT_mo_HMOX1F	*HMOX1*	CAG TCG CCT CCA GAG TTT CC	284
qRT_mo_HMOX1R		TAC AAG GAA GCC ATC ACC AGC	
qRT_mo_GCL-MF	*GCLM*	CTG CAA AAC TGT TCA TTG TAG G	280
qRT_mo_GCL-MR		CTA TTG GGT TTT ACC TGT G	
qRT_mo_NFE2L2F	*NFE2L2*	GCA ACT CCA GAA GGA ACA GG	142
qRT_mo_NFE2L2R		GTG GGC AAC CTG GGA GTA G	
moPrP_103F	*PRNP*	AAT CAG TGG AAC AAG CCC AG	103
moPrP_103R		CCA GCA TGT AGC CAC CAA G	
mo_actin_Alt-F	*ACTB*	CAG CTT CTT TGC AGC TCC TT	375
mo_actin_Alt-R		CAC GAT GGA GGG GAA TAC AG	

## Data Availability

Data is contained within the article and supplementary material.

## Supplementary Materials

The following supporting information can be downloaded at: https://www.mdpi.com/article/10.3390/antiox11040726/s1, Figure S1: cell viability assessment by means of the 3-(4,5-dimethylthiazol-2-yl)-2,5-diphenyl-2H-tetrazolium bromide (MTT) assay for CA (A) and CS (B) following 48 h incubation in N2a58 and N2a22L cells; Figure S2: Western blot of N2a58 and N2a22L cell lysates plus (PK+) or minus PK (PK−) proteinase K treatment for PrP detection; Figure S3: Control RT-QuiC reactions.
