# Supplementary material for: Carnosic Acid and Carnosol Display Antioxidant and Anti-Prion Properties in In Vitro and Cell-Free Models of Prion Diseases
Source: Antioxidants (Basel). 2022 Apr 6;11(4):726. doi: 10.3390/antiox11040726 (PMC9027925; doi:10.3390/antiox11040726)
Supplement: Supplementary file 1 [file antioxidants-11-00726-s001.zip › antioxidants-1641047-Supplementary.pdf]

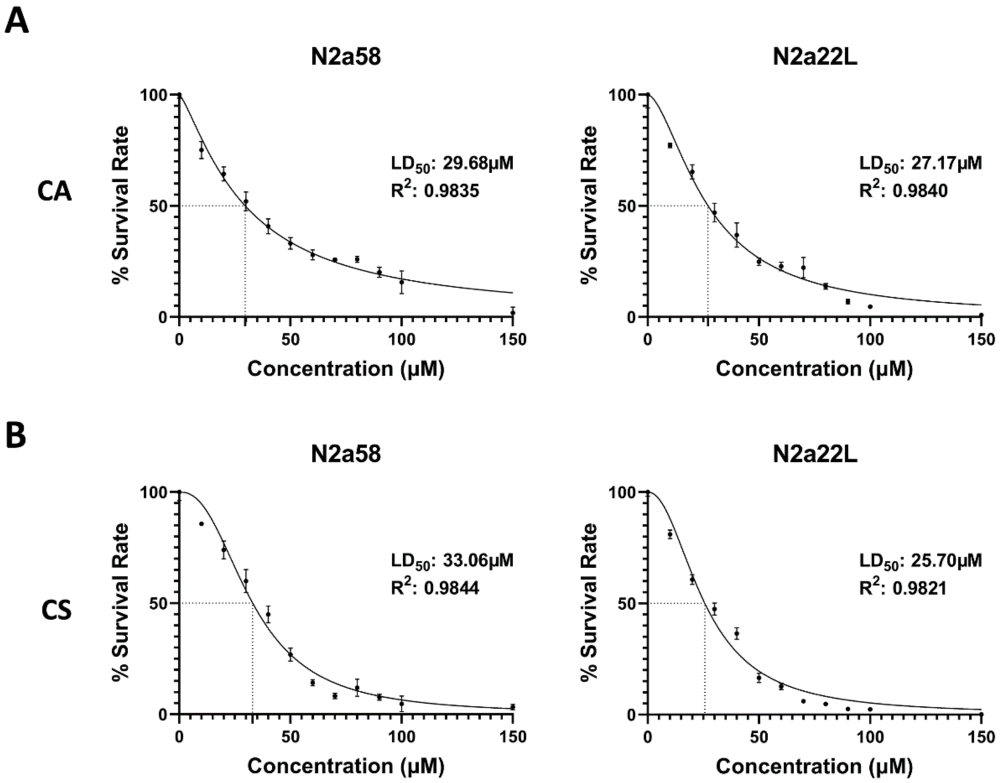

Supplementary Figure S1. Cell viability assessment by means of the 3-(4,5-dimethylthiazol-2-yl)-2,5-diphenyl-2H-tetrazolium bromide (MTT) assay for CA (**A**) and CS (**B**) following 48 h incubation in N2a58 and 22LN2a58 cells. Graphs depict the absorbance at 570 nm determined for each cell line following treatment with the indicated compounds at different concentrations. The background absorbance of the plates at 630 nm was also measured and subtracted from 570 nm measurement. LD_{50} was estimated for each compound and cell line based on non-linear regression analysis for curve fitting using the GraphPad software (v 8.0). The black dots in graphs A and B for CA and CS represent the fitted curve in each case; determined LD_{50} values are depicted in each graph. The R^2 values indicate the goodness of the fit.

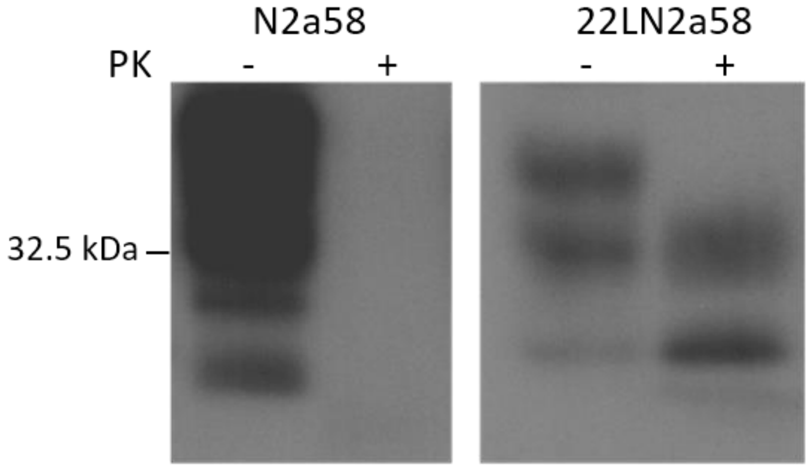

Supplementary Figure S2. Western blot of N2a58 and 22LN2a58 cell lysates plus (PK+) or minus PK (PK-) proteinase K treatment for PrP detection. In N2a58 lysates PrPC is detected in the PK- lysate, while it is completely digested following PK treatment. When 22LN2a58 lysates are similarly processed (PK +: 1.25 µg PK/mg total protein) only the partially resistant PrPSc fraction is detected.

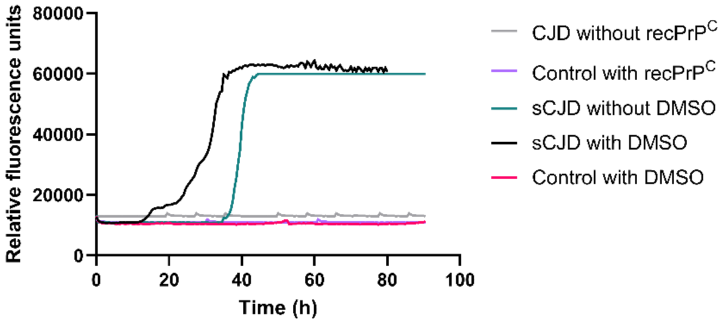

Supplementary Figure S3. RT-QuIC assays controls. Diverse control RT-QuiC reactions were set up using CSF from non-sCJD or CJD patients, in the absence or presence of recPrP^C and/or DMSO. Whenever DMSO was added, its final concentration was 2% v/v. In all cases the seeding response curve of a single CSF sample was graphed. The graphs correspond to the reactions summarized in the following table:

Seed CSF	***Recombinant PrP***	***DMSO (2% v/v)***	***Label***
sCJD	*-*	*+*	*sCJD without recPrP^C*
non-sCJD	*+*	*+*	*Control with recPrP^C*
sCJD	*+*	*-*	*sCJD without DMSO*
sCJD	*+*	*+*	*sCJD with DMSO*
non-sCJD	*+*	*-*	*Control with DMSO*

Very low Th-T fluorescence readings were recorded in the absence of sCJD-CSF or recPrP^C, indicating that very few PrP aggregates are generated. However, when both sCJD-CSF and recPrP^C are present, aggregates are formed. In this case, the presence of DMSO does not significantly alter the formation of the aggregates.
